# Supplementary material for: A Humanized Yeast Phenomic Model of Deoxycytidine Kinase to Predict Genetic Buffering of Nucleoside Analog Cytotoxicity
Source: Genes (Basel). 2019 Sep 30;10(10):770. doi: 10.3390/genes10100770 (PMC6826991; doi:10.3390/genes10100770)
Supplement: Supplementary file 1 [file genes-10-00770-s001.zip › Additional_Files/Additional_File7_GOTermHeatmaps.zip!/Additional_File7_GOTermHeatmaps/DNA-dependent_DNA_replication.pdf]

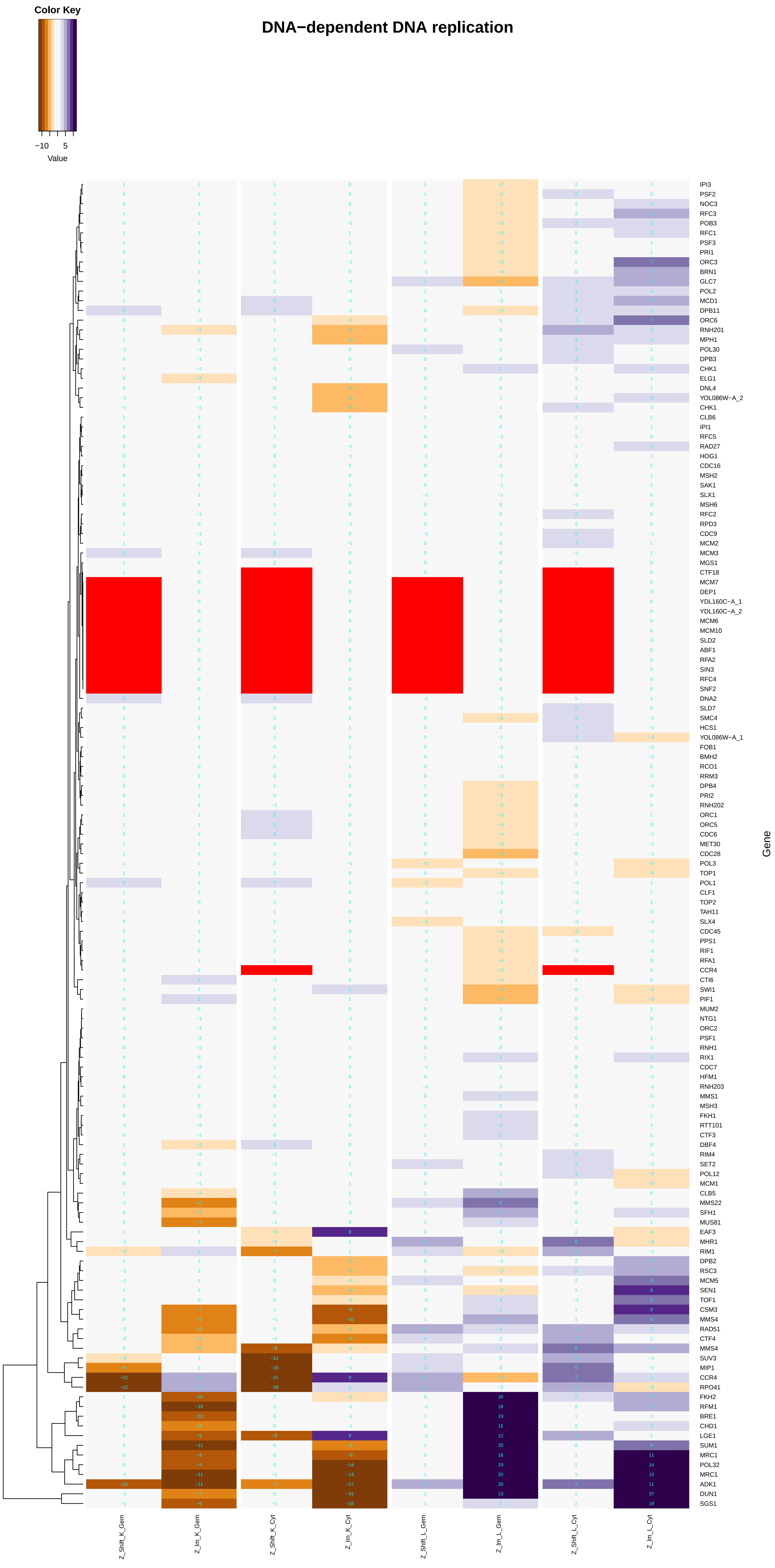

DNA−dependent DNA replication
Color Key
−10
5
Value
Gene
IPI3
PSF2
NOC3
RFC3
POB3
RFC1
PSF3
PRI1
ORC3
BRN1
GLC7
POL2
MCD1
DPB11
ORC6
RNH201
MPH1
POL30
DPB3
CHK1
ELG1
DNL4
YOL086W−A_2
CHK1
CLB6
IPI1
RFC5
RAD27
HOG1
CDC16
MSH2
SAK1
SLX1
MSH6
RFC2
RPD3
CDC9
MCM2
MCM3
MGS1
CTF18
MCM7
DEP1
YDL160C−A_1
YDL160C−A_2
MCM6
MCM10
SLD2
ABF1
RFA2
SIN3
RFC4
SNF2
DNA2
SLD7
SMC4
HCS1
YOL086W−A_1
FOB1
BMH2
RCO1
RRM3
DPB4
PRI2
RNH202
ORC1
ORC5
CDC6
MET30
CDC28
POL3
TOP1
POL1
CLF1
TOP2
TAH11
SLX4
CDC45
PPS1
RIF1
RFA1
CCR4
CTI6
SWI1
PIF1
MUM2
NTG1
ORC2
PSF1
RNH1
RIX1
CDC7
HFM1
RNH203
MMS1
MSH3
FKH1
RTT101
CTF3
DBF4
RIM4
SET2
POL12
MCM1
CLB5
MMS22
SFH1
MUS81
EAF3
MHR1
RIM1
DPB2
RSC3
MCM5
SEN1
TOF1
CSM3
MMS4
RAD51
CTF4
MMS4
SUV3
MIP1
CCR4
RPO41
FKH2
RFM1
BRE1
CHD1
LGE1
SUM1
MRC1
POL32
MRC1
ADK1
DUN1
SGS1
Z_Shift_K_Gem
Z_lm_K_Gem
Z_Shift_K_Cyt
Z_lm_K_Cyt
Z_Shift_L_Gem
Z_lm_L_Gem
Z_Shift_L_Cyt
Z_lm_L_Cyt

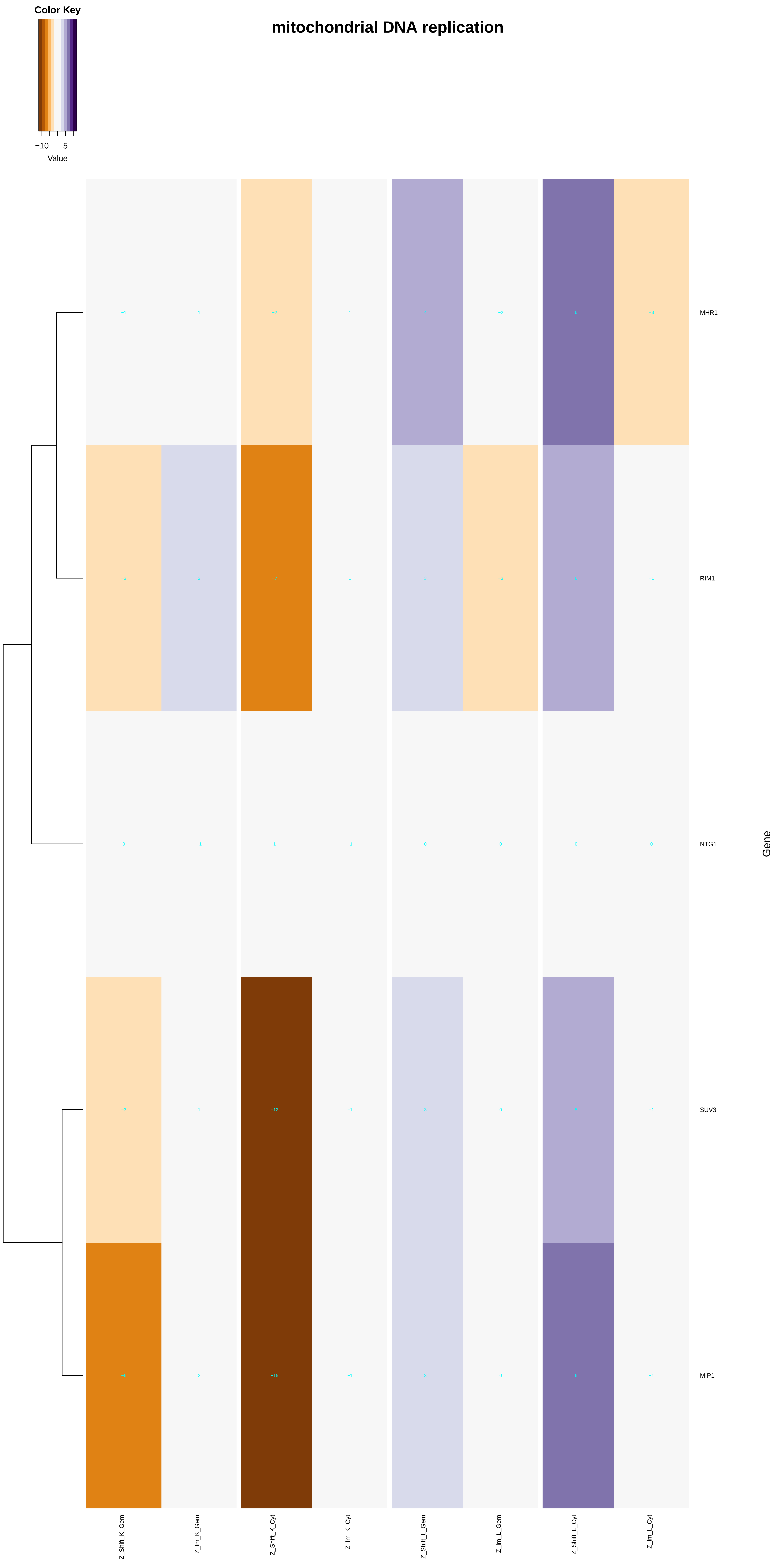
Color Key
-10
5
Value
mitochondrial DNA replication
MHR1
RIM1
NTG1
SUV3
MIP1
Gene
Z_Shift_K_Gem
Z_Im_K_Gem
Z_Shift_K_Cyt
Z_Im_K_Cyt
Z_Shift_L_Gem
Z_Im_L_Gem
Z_Shift_L_Cyt
Z_Im_L_Cyt

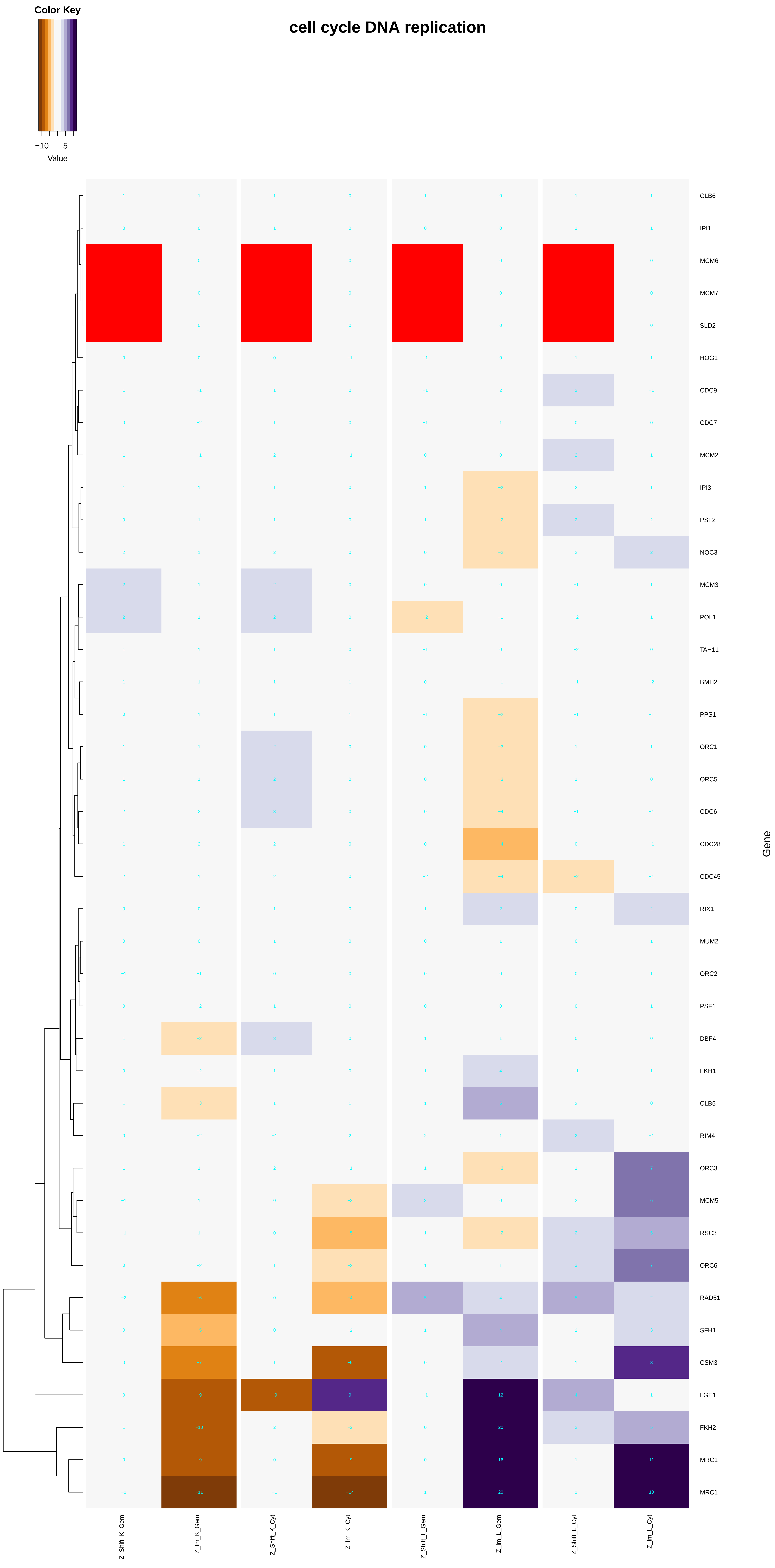

cell cycle DNA replication
Color Key
−10
5
Value
Gene
CLB6
IPI1
MCM6
MCM7
SLD2
HOG1
CDC9
CDC7
MCM2
IPI3
PSF2
NOC3
MCM3
POL1
TAH11
BMH2
PPS1
ORC1
ORC5
CDC6
CDC28
CDC45
RIX1
MUM2
ORC2
PSF1
DBF4
FKH1
CLB5
RIM4
ORC3
MCM5
RSC3
ORC6
RAD51
SFH1
CSM3
LGE1
FKH2
MRC1
MRC1
Z_Shift_K_Gem
Z_lm_K_Gem
Z_Shift_K_Cyt
Z_lm_K_Cyt
Z_Shift_L_Gem
Z_lm_L_Gem
Z_Shift_L_Cyt
Z_lm_L_Cyt

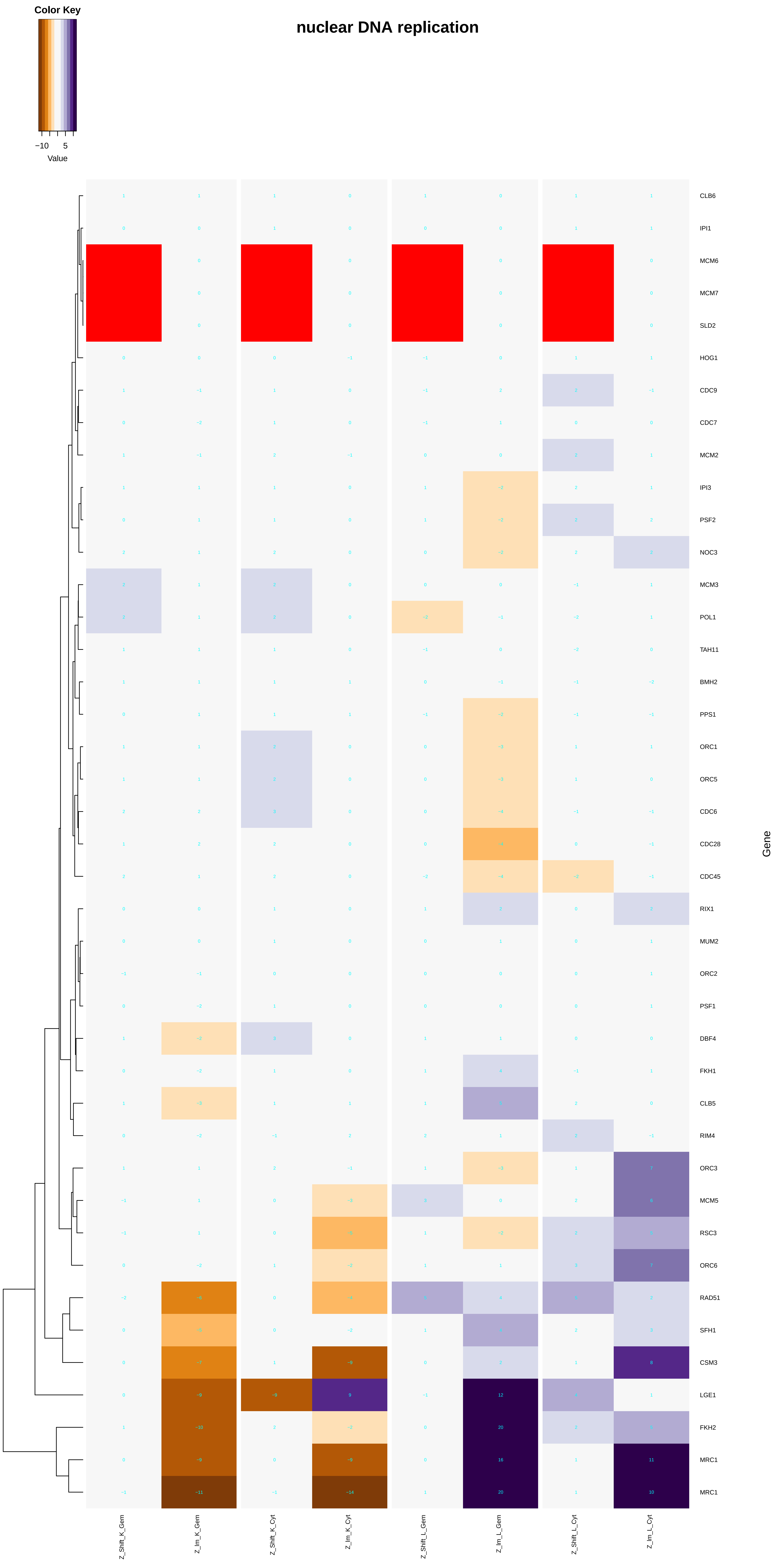
nuclear DNA replication
Color Key
−10 5
Value
CLB6
IPI1
MCM6
MCM7
SLD2
HOG1
CDC9
CDC7
MCM2
IPI3
PSF2
NOC3
MCM3
POL1
TAH11
BMH2
PPS1
ORC1
ORC5
CDC6
CDC28
CDC45
RIX1
MUM2
ORC2
PSF1
DBF4
FKH1
CLB5
RIM4
ORC3
MCM5
RSC3
ORC6
RAD51
SFH1
CSM3
LGE1
FKH2
MRC1
MRC1
Gene
Z_Shift_K_Gem
Z_Im_K_Gem
Z_Shift_K_Cyt
Z_Im_K_Cyt
Z_Shift_L_Gem
Z_Im_L_Gem
Z_Shift_L_Cyt
Z_Im_L_Cyt

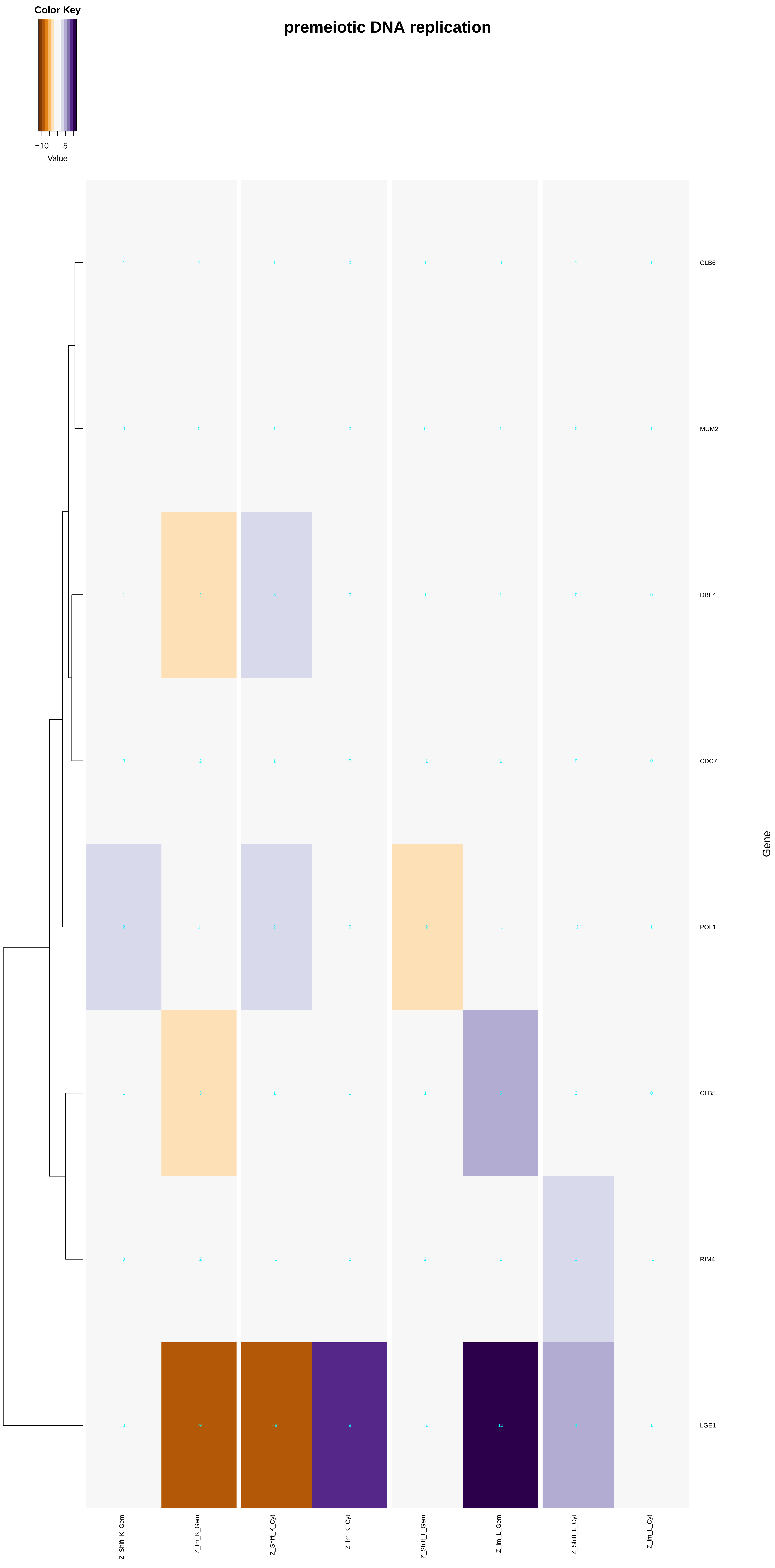
Color Key
−10
5
Value
premeiotic DNA replication
CLB6
MUM2
DBF4
CDC7
POL1
CLB5
RIM4
LGE1
Gene
Z_Shift_K_Gem
Z_Im_K_Gem
Z_Shift_K_Cyt
Z_Im_K_Cyt
Z_Shift_L_Gem
Z_Im_L_Gem
Z_Shift_L_Cyt
Z_Im_L_Cyt

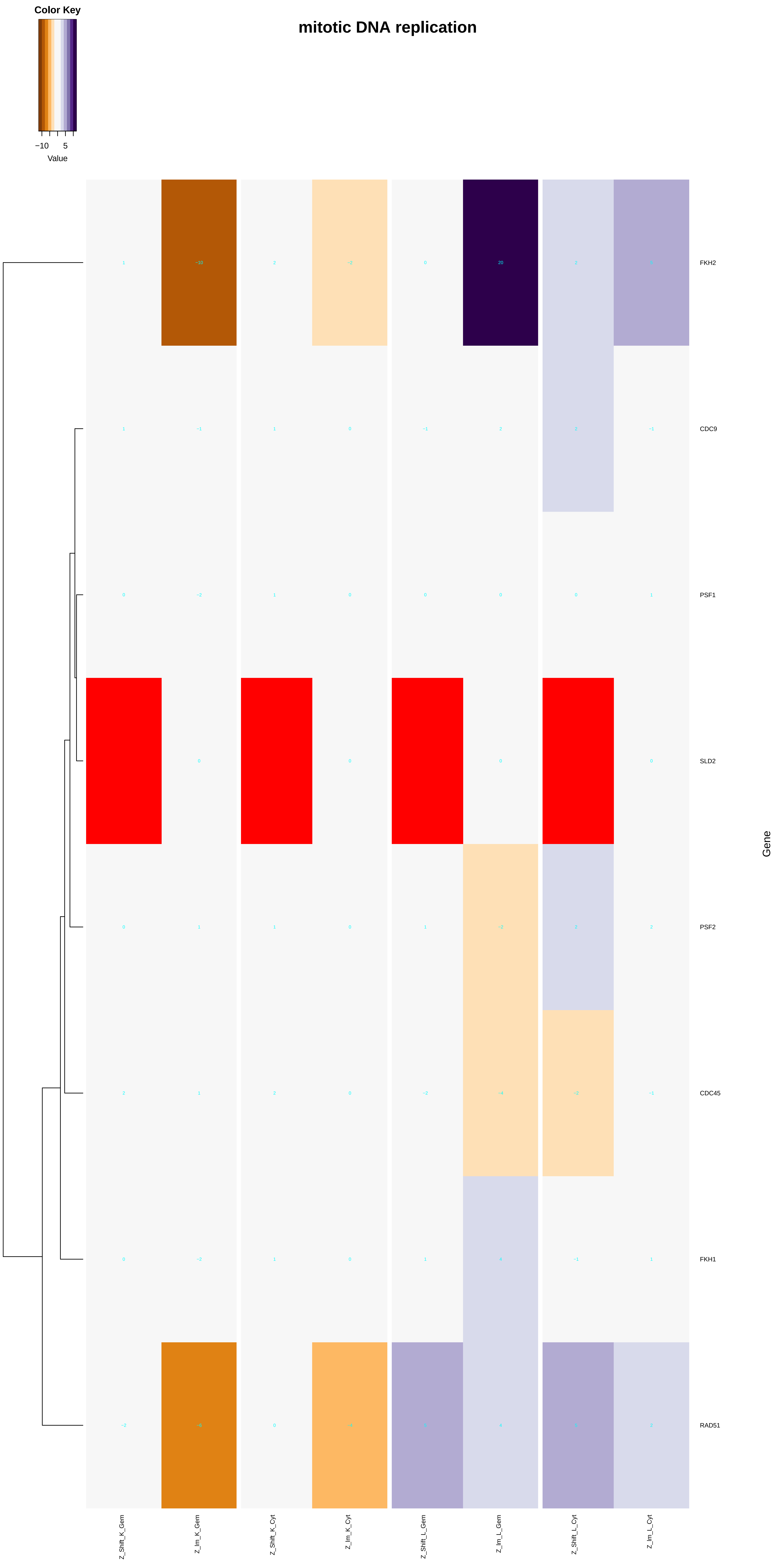
mitotic DNA replication
Color Key
−10
5
Value
FKH2
CDC9
PSF1
SLD2
PSF2
CDC45
FKH1
RAD51
Gene
Z_Shift_K_Gem
Z_Im_K_Gem
Z_Shift_K_Cyt
Z_Im_K_Cyt
Z_Shift_L_Gem
Z_Im_L_Gem
Z_Shift_L_Cyt
Z_Im_L_Cyt
